# Immune checkpoint proteins are associated with persistently high liver stiffness after successful HCV treatment in people with HIV: a retrospective study

**DOI:** 10.3389/fimmu.2024.1505864

**Published:** 2024-12-17

**Authors:** Rubén Martín-Escolano, Ana Virseda-Berdices, Juan Berenguer, Juan González-García, Oscar Brochado-Kith, Amanda Fernández-Rodríguez, Cristina Díez, Victor Hontañon, Salvador Resino, María Ángeles Jiménez-Sousa

**Affiliations:** ^1^ Unidad de Infección Viral e Inmunidad, Centro Nacional de Microbiología (CNM), Instituto de Salud Carlos III (ISCIII), Majadahonda, Madrid, Spain; ^2^ Centro de Investigación Biomédica en Red en Enfermedades Infecciosas (CIBERINFEC), Instituto de Salud Carlos III (ISCIII), Madrid, Spain; ^3^ Unidad de Enfermedades Infecciosas/VIH, Hospital General Universitario “Gregorio Marañón”, Madrid, Spain; ^4^ Instituto de Investigación Sanitaria Gregorio Marañón (IiSGM), Madrid, Spain; ^5^ Servicio de Medicina Interna-Unidad de VIH, Hospital Universitario La Paz, Madrid, Spain; ^6^ Instituto de Investigación Sanitaria La Paz (IdiPAZ), Madrid, Spain

**Keywords:** HIV/HCV-coinfection, cirrhosis, antiviral therapy, liver stiffness, biomarkers

## Abstract

Various immune checkpoint proteins have been linked to cirrhosis. This study aimed to explore the association between plasma levels of these proteins measured one year after successful HCV treatment and persistently liver stiffness (defined as liver stiffness measurement (LSM) ≥ 12.5 kPa) five years after HCV treatment in people with HIV (PWH). We conducted a retrospective study involving 39 patients with HIV/HCV-coinfection who had advanced fibrosis or cirrhosis and achieved sustained virologic response (SVR). Plasma samples were obtained one year after treatment, and levels of immune checkpoints along with inflammatory biomarkers were evaluated using a Luminex 200^TM^ analyzer. Statistical analyses were performed using Generalized Linear Models (GLMs) with a gamma distribution. Spearman correlation tests were used to analyze the correlation between significant immune checkpoints and inflammatory biomarkers. Although LSM values showed a decreasing trend over the years following successful HCV treatment, this trend was not statistically significant due to substantial variability among PWH. Persistently high liver stiffness was observed in 61.5% of patients five years after HCV treatment. Elevated plasma levels of soluble BTLA, PD-1, and TIM-3 one year after HCV treatment were associated with persistently liver stiffness five years later. These significant immune checkpoints were found to correlate with inflammatory biomarkers in PWH with persistently high liver stiffness. In conclusion, increased plasma concentrations of immune checkpoints one year after successful HCV therapy were linked to persistently high liver stiffness five years later, particularly BTLA, PD-1, and TIM-3. This suggests a potential immunopathological mechanism in ongoing liver stiffness post-HCV eradication.

## 1 Introduction

Direct-acting antivirals (DAA) have revolutionized the therapy of chronic hepatitis C with rates of sustained virologic response (SVR) higher than 90%, even in patients previously considered as difficult to treat, such as those with decompensated liver disease or with HIV infection (1).

Clinical studies have shown that SVR following DAA therapy improves clinical outcomes among patients with HCV-related compensated cirrhosis. However, a small proportion persists at risk of developing liver-related events. Successful DAA therapy decreased portal pressure among patients with HCV-related cirrhosis with or without HIV; nonetheless, the frequent persistence of clinically significant portal hypertension indicates a persistent risk of clinical progression or death (2, 3).

Therefore, it is vital to investigate the pathophysiological mechanisms involved in the evolution of liver disease among patients with hepatitis C and advanced fibrosis or cirrhosis with cleared HCV infection. There is evidence that HCV may induce epigenetic and transcriptional changes in the liver tissue associated with hepatocellular carcinoma (HCC) that persist following SVR to DAA therapy (4). Besides, several immune checkpoint proteins are upregulated during acute and chronic infections, and different studies have demonstrated an association between immune checkpoint proteins and clinical outcomes in both HCV and HIV infection (5–11). In HCV infection, surface immune checkpoints have been correlated with disease progression and poor prognosis (12–16). Signaling via these proteins can drive effector immune T cells into a state known as “exhaustion”, contributing to reduced effector function, sustained expression of immune checkpoint molecules, poor recall responses, limited immune clearance of pathogens, escape from immune control, and disease progression (17). Similarly, surface immune checkpoints are also observed upregulated in HIV infection on both CD4+ and CD8+ T cells, and correlated with disease progression as reflected in decreased T cell function, decreased CD4+ T cell counts, increased viral RNA replication, and HIV reservoir enrichment (18). After HCV treatment, it has been observed that surface immune checkpoint proteins decline but remain elevated compared to those of healthy people (19), regardless treatment is started early or late after infection (20). Alternatively, increase in liver cirrhosis severity has been associated with a skewed immune profile, characterized by increased immune checkpoint proteins and systemic inflammation, leading to inadequate immune protection (5).

In recent studies conducted within the Marathon study, we have showed that increased plasma levels of soluble immune checkpoint proteins such as B and T lymphocyte attenuator (BTLA), cluster of differentiation 137 (CD137) (4-1BB), CD80, glucocorticoid-induced TNFR-related protein (GITR), lymphocyte activation gene-3 (LAG-3) and programmed death-ligand 1 (PD-L1) before HCV therapy were associated with a long-term increase in hepatic steatosis index (HSI) after successful therapy, suggesting a potential predictive role of these markers for early detection of progression towards steatosis in these patients (21). Alternatively, lower baseline levels of soluble BTLA and LAG-3 before HCV therapy were significantly linked to an increased risk of developing metabolic disorders after treatment, underscoring the relevance of these markers in metabolic outcomes (22). However, no studies have analyzed the role of immune checkpoint proteins in liver disease after successful HCV therapy in people with HIV (PWH).

Our objective was to assess the association between plasma levels of immune checkpoint proteins one year after successful HCV therapy and persistently high liver stiffness (defined as a liver stiffness measurement (LSM) ≥ 12.5 kPa) five years later in PWH with advanced fibrosis or cirrhosis.

## 2 Methods

### 2.1 Study subjects

This retrospective study included patients with HIV/HCV-coinfection who had advanced fibrosis or cirrhosis and successfully cleared HCV infection following interferon (IFN)-based therapy (either pegylated interferon α (peg-IFN-α) combined with ribavirin or peg-IFN-α with ribavirin and direct-acting antivirals (DAAs)) or IFN-free DAA therapy conducted between 2012 and 2021 across 10 centers in Spain. Participants were selected from the GeSIDA 10318 cohort (Marathon Study Group; Appendix 1). All patients achieved SVR, defined as undetectable HCV-RNA levels 12 to 24 weeks after completing anti-HCV treatment, depending on the treatment regimen, and maintained SVR throughout follow-up. They also had clinical data and frozen plasma samples available one year after successful HCV treatment, along with additional clinical information collected about five years later. All subjects were on stable antiretroviral therapy (ART) for over six months, with an undetectable plasma HIV viral load (<50 copies/mL). Patients with a history of HCV reinfection, co-infection with hepatitis B virus (HBV), acute hepatitis C, hepatocellular carcinoma (HCC), or hepatic decompensation were excluded from the study.

The Research Ethics Committee of the Institute of Health Carlos III (CEI PI 72_2021) approved the study, which was carried out in compliance with the principles of the Declaration of Helsinki. All individuals involved provided written informed consent prior to their participation.

### 2.2 Clinical data and samples

We leveraged the GeSIDA 10318 cohort database to obtain the clinical data for this investigation. From each patient, peripheral blood was collected in EDTA tubes by venipuncture and sent to the HIV BioBank (23). Plasma aliquots were obtained by centrifugation and were stored frozen (-80°C) until use.

### 2.3 Outcome variable

The outcome variable was a persistently liver stiffness (LSM ≥ 12.5 kPa) about five years after completing HCV treatment evaluated by transient elastography using FibroScan devices. We chose this cutoff value because it has been found to be associated with a high likelihood of liver cirrhosis (24).

### 2.4 Multiplex immunoassays

An Immuno-Oncology Checkpoint 14-Plex Human ProcartaPlex™ Panel 1 (InvitrogenTM) was used to quantify various plasma-soluble proteins using a Luminex 200™ analyzer (Luminex Corporation, Austin, TX, USA) following the manufacturer’s protocol. The proteins measured included: BTLA, cytotoxic T-lymphocyte-associated protein 4 (CD152/CTLA-4), CD27, CD28, CD80, CD137 (4-1BB), GITR, herpesvirus entry mediator (HVEM), indoleamine 2,3-dioxygenase (IDO), LAG-3, programmed cell death protein 1 (PD-1), PD-L1, programmed death-ligand 2 (PD-L2), and T-cell immunoglobulin and mucin-domain containing-3 (TIM-3).

Additionally, a ProcartaPlex™ multiplex assay (Invitrogen™) was used to quantify a range of anti-inflammatory/suppressor protein markers. The proteins measured included: interleukin 8 (IL-8), IL-18, IL-1 receptor antagonist (IL-1RA), interferon-inducible protein 10 (IP-10), monocyte chemoattractant protein-1 (MCP-1), and endothelial dysfunction indicators like tumor necrosis factor receptor-1 (TNF-RI).

Raw fluorescence intensity (FI) values, expressed in arbitrary units (a.u.), were used for analysis.

### 2.5 Statistical analysis

For descriptive analysis, categorical variables were shown as absolute count (percentage) and quantitative variables as median (interquartile range, IQR). Comparisons between groups were conducted using the Chi-square and Mann-Whitney U tests for categorical and quantitative variables, respectively. The Wilcoxon signed range test was used to compare paired data (repeated measures).

To evaluate the association between plasma levels of immune checkpoint proteins (dependent variable) and persistently high liver stiffness (LSM ≥ 12.5 kPa; independent variable) about five years later, Generalized Linear Models (GLMs) with a gamma distribution (log-link) were used. The analysis provided the arithmetic mean ratio (AMR), along with its 95% confidence interval (95% CI) and significance level. Multiple testing corrections were applied using the Benjamini and Hochberg method, and biomarkers with a p-value < 0.05 (two-tailed) and a q-value < 0.10 were considered statistically significant. Next, significant biomarkers from univariate analysis were analyzed by GLMs adjusted for the most relevant characteristics [age, gender, HCV treatment (IFN-based therapy or DAAs), LSM at one year after treatment, and time elapsed between the two point times (time elapsed between the sample collection, taken one year after successful HCV treatment, and the LSM clinical data (end of follow-up), about five years after HCV treatment)], which were previously selected by a stepwise method (forward) according to the lowest Akaike information criteria (AIC) for each model.

Next, we analyzed the diagnostic performance of significant metabolites resulting from adjusted GLM model for predicting persistently elevated LSM using the area under the receiver-operating characteristic (AUROC) curve.

Spearman correlation tests were used to analyze the correlation between significant immune checkpoint proteins and inflammatory biomarkers. Correlations were deemed relevant if they had a coefficient of r > 0.30 or r < -0.30 and met the criteria for statistical significance (p < 0.05; q-value < 0.10).

All statistical analyses were conducted using the R software package (version 4.2.0, R Foundation for Statistical Computing, Vienna, Austria).

## 3 Results

### 3.1 Patient characteristics

The baseline characteristics of 39 patients with HIV/HC-coinfection prior to starting HCV therapy are shown in **Table 1**. Overall, the median age was 51, 79.5% were male, and the body mass index (BMI) was 24.6 kg/m^2^. Regarding addictive substance use, 61.5% were current smokers, and 76.9% and 42.6% had a prior history of injection drug use and alcohol intake, respectively. Regarding liver markers, the median LSM was 26.0 kPa, HSI was 34.0, and FIB-4 was 3.4. Regarding the virological aspects, 28 (75.7%) patients were infected with HCV genotype 1, and the CD4^+^ T cell count was 446 cells/mm^3^.

**Table 1:** Baseline characteristics of HIV/HCV-coinfected patients before HCV therapy.

	All patients
No.	39
Age (years)	51 (48–53)
Gender (male)	31 (79.5%)
BMI ((kg/m^2^)	24.6 (22.7–26.4)
Smoker	
Never	5 (12.8%)
Previous (>6 months)	10 (25.6%)
Current	24 (61.5%)
Alcohol intake (>50g/day)	
Never	19 (48.7%)
Previous (>6 months)	18 (46.2%)
Current	2 (5.1%)
Intravenous drug user	
Never	9 (23.1%)
Previous (>6 months)	30 (76.9%)
Current	0 (0%)
Previous HCV therapy	21 (53.8%)
MASLD (n=37)	8 (21.6%)
Liver markers	
LSM (kPa)	26.0 (15.6–34.6)
HSI (n = 37)	34.0 (29.8–35.6)
FIB-4 (n = 37)	3.4 (2.4–7.5)
HCV genotype (n = 37)	
1	28 (75.7%)
3	5 (13.5%)
4	4 (10.8%)
Log_10_ HCV-RNA (IU/mL)	6.1 (5.6–6.5)
HCV-RNA > 850.000 IU/mL	22 (56.4%)
HCV therapy	
pegIFN	22 (56.4%)
DAAs	17 (43.6%)
HIV markers	
Previous AIDS (n = 38)	1 (2.6%)
CD4+ T-cells/mm^3^	446.0 (262.5–664.0)
CD4+ T-cells < 500 cells/mm^3^	25 (64.1%)
HIV antiretroviral therapy	
NRTI + NNRTI	11 (28.2%)
NRTI + II	15 (38.5%)
NRTI + PI	6 (15.4%)
PI+II+NNRTI/MVC	1 (2.6%)
Others	6 (15.4%)

Statistics: The data are presented as absolute numbers (percentages) and medians (interquartile range). Abbreviations: AIDS, acquired immune deficiency syndrome; BMI, body mass index; FIB-4, fibrosis-4; DAAs, direct-acting antivirals; HCV, hepatitis C virus; HIV, human immunodeficiency virus; HSI, hepatic steatosis index; II, HIV integrase inhibitor; kPa, kilopascal; LSM, liver stiffness measurement; MASLD, metabolic dysfunction-associated steatotic liver disease; NNRTI, non-nucleoside analogue HIV reverse transcriptase inhibitor; NRTI, nucleoside analogue HIV reverse transcriptase inhibitor; pegIFN, pegylated interferon; PI, HIV protease inhibitor.

### 3.2 Variation in LSM

**Figure 1:**
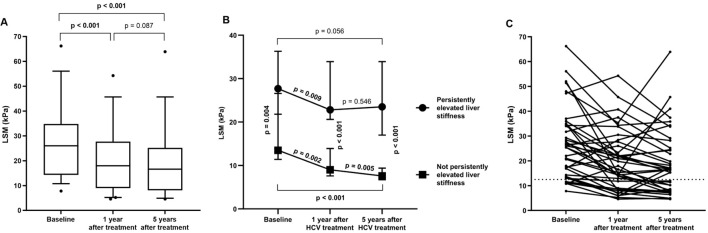
**(A)** Evolution of LSM values from baseline to five years (end of follow-up) after completion of successful HCV treatment in HIV/HCV-coinfected patients. Statistics: Data were calculated by the Wilcoxon test. **(B)** Evolution of LSM values from baseline to five years (end of follow-up) after completion of successful HCV treatment in HIV/HCV-coinfected patients stratifying by persistently elevated liver stiffness. Statistics: Data were calculated by the Mann-Whitney and Wilcoxon tests. **(C)** Individual evolution of LSM values from one year to five years (end of follow-up) after completion of successful HCV treatment in HIV/HCV-coinfected patients.

Overall, there was a significant reduction (p< 0.001) in LSM value from baseline (before HCV treatment) (26.0 (15.6–34.6) kPa) to one year after the completion of HCV treatment (18.0 (10.4–26.9) kPa), and a downward trend (p=0.087) from one year to five years after HCV treatment (16.6 (8.4–24.9) kPa) (**Figure 1A**).

Next, when considering the persistence of elevated LSM during the follow-up period (five years after the successful completion of HCV treatment), 24 (61.5%) PWH had persistently elevated LSM. These individuals showed similar baseline characteristics to those without persistent LSM, except for gender (p= 0.036), LSM (p= 0.004), and type of HCV treatment (p= 0.007) (**Supplementary Table 1**).

Regarding LSM evolution, PWH with persistently elevated LSM showed an LSM of 27.7 (21.8–36.3) kPa, 22.8 (20.6–33.9) kPa and 23.5 (17.0–33.9) kPa at baseline, one and five years after HCV treatment, respectively, versus 13.5 (11.4–26.6) kPa, 9.0 (7.6–13.9) kPa and 7.6 (6.7–9.4) kPa for those PWH without persistent LSM (**Figure 1B**). Although significant reductions (p= 0.009 and p= 0.002) in LSM values were observed from baseline to one year after HCV treatment in both groups (**Figure 1B**), a significant reduction (p= 0.005) was only found from one year to five years after HCV treatment in PWH with not persistently high LSM. No significant difference (p= 0.546) was observed in PWH with persistently high LSM. **Figure 1C** shows the variability between patients.

### 3.3 Plasma biomarkers related to persistently liver stiffness

**Figure 2:**
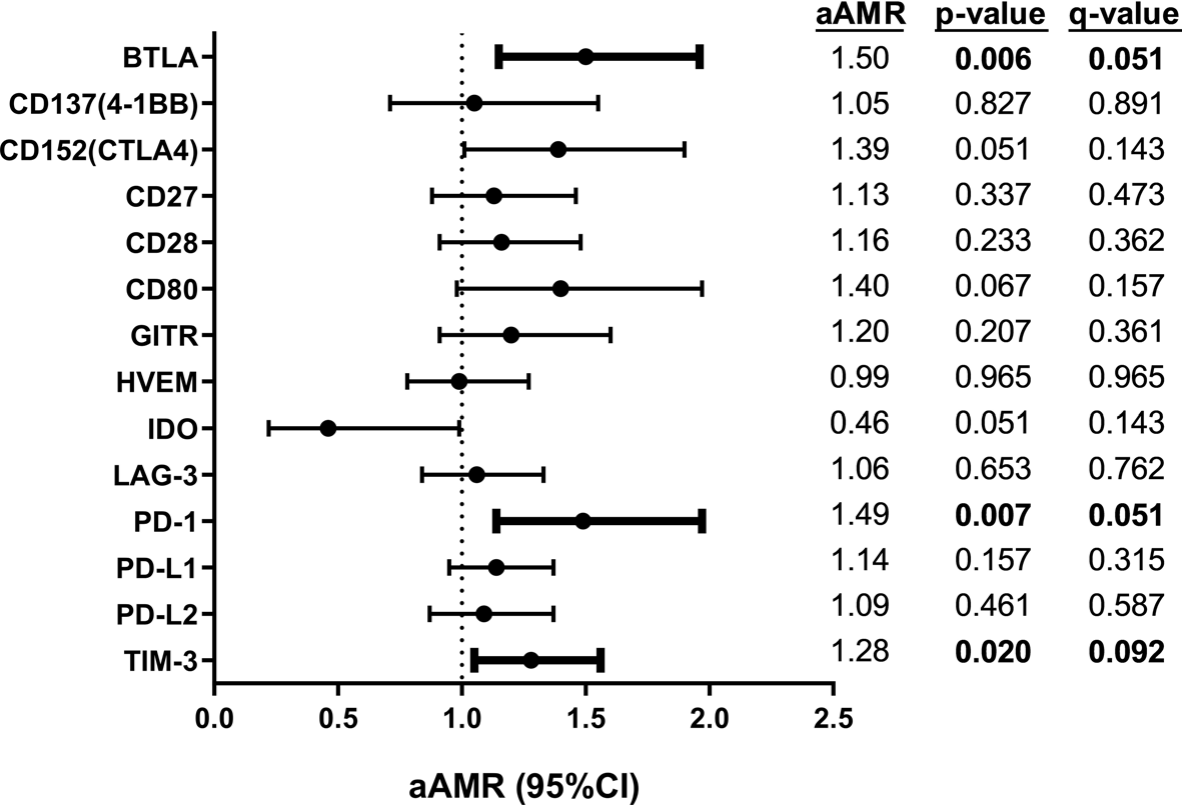
Association of plasma immune checkpoint proteins one year after completion of successful HCV treatment with persistently high liver stiffness (LSM ≥12.5 kPa) to the end of follow-up (five years after treatment) in HIV/HCV-coinfected patients. Statistics: Data were calculated by Generalized Linear Models (GLM) with a gamma distribution (log-link), adjusted by age, gender, HCV treatment (IFN-based therapy or DAAs), LSM at 36 weeks after SVR, and time from SVR to follow-up time (see Materials and Methods Section). The q-values represent p-values corrected for multiple testing using the False Discovery Rate (FDR). Statistically significant differences are shown in bold. AMR, arithmetic mean ratio; aAMR, adjusted AMR; 95%CI, 95% of confidence interval; p, level of significance; q, corrected level of significance; BTLA, B, and T lymphocyte attenuator; CD, cluster of differentiation; GITR, glucocorticoid-induced TNFR-related; HVEM, herpesvirus entry mediator; IDO, indoleamine 2,3-dioxygenase; LAG-3, lymphocyte activation gene-3; LSM, liver stiffness measurement; PD-1, programmed cell death protein 1; PD-L1, programmed death-ligand 1; PD-L2, programmed death-ligand 2; TIM-3, T-cell immunoglobulin and mucin-domain containing-3.

Unadjusted GLM models showed significant direct associations between plasma levels of soluble BTLA, CD80, LAG-3, PD-1, PD-L2, and TIM-3 one year after completion of successful HCV treatment and the presence of persistently liver stiffness about five years after the completion of HCV treatment (**Supplementary Table 2**). In GLM models adjusted by main epidemiological and clinical characteristics, we only found significant direct associations for three immune checkpoint proteins (**Figure 2**; full description in **Supplementary Table 2**): BTLA (aAMR= 1.49; p= 0.006), PD-1 (aAMR= 1.49; p= 0.007), and TIM-3 (aAMR= 1.28; p= 0.020). However, their predictive performance was suboptimal (AUROC <0.70).

Notably, two of these proteins, PD-1 and TIM-3, also showed significant direct associations at baseline (before HCV treatment) with persistently elevated liver stiffness about five years after the completion of HCV treatment: PD-1 (aAMR= 2.71; p= 0.014), and TIM-3 (aAMR= 1.26; p= 0.048) (**Supplementary Table 3**).

### 3.4 Correlation analysis between immune checkpoint proteins and inflammatory biomarkers

**Figure 3:**
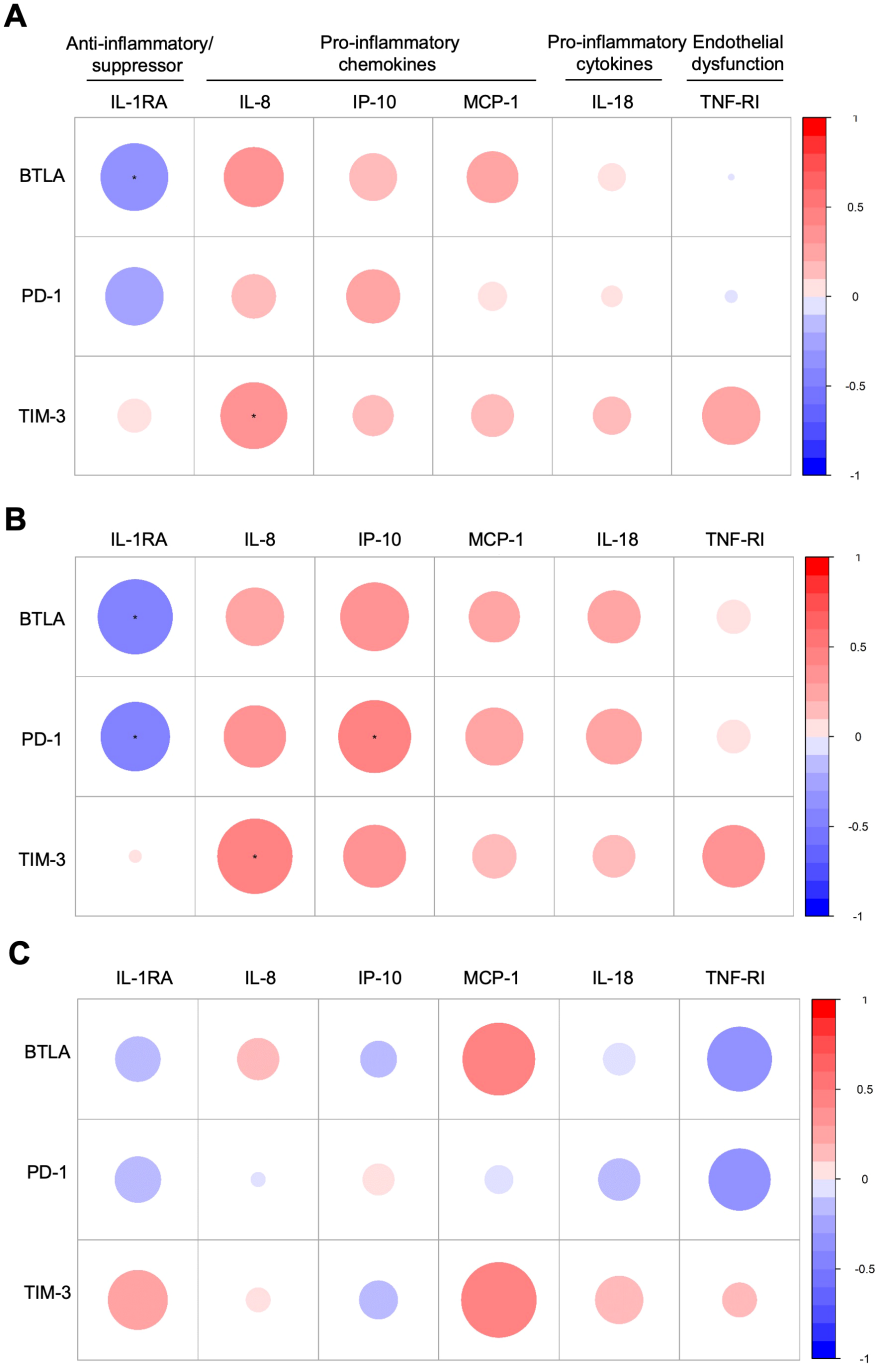
Spearman correlation plot between significant immune checkpoint proteins and inflammatory biomarkers: **(A)** all people with HIV (PWH); **(B)** PWH who had persistently high liver stiffness (LSM ≥ 12.5 kPa) five years after the successful completion of HCV treatment: **(C)** PWH who had not persistently high liver stiffness (LSM < 12.5 kPa) five years after the successful completion of HCV treatment. The size of the circles is proportional to the strength of the correlation, and the color represents the direction (color legends are shown on the right), where large dark blue represents a strong negative correlation, and a large dark red circle represents a strong positive correlation. Inflammatory biomarkers are on the horizontal axis, and immune checkpoint proteins are on the vertical axis. Those correlations with rho>0.30 o rho<-0.30, p-valor<0.05, and q-valor<0.10 are shown with an asterisk. BTLA, B, and T lymphocyte attenuator; PD-1, programmed cell death protein 1; TIM-3, T-cell immunoglobulin and mucin-domain containing-3; IL, interleukin; IL-1RA, IL-1 receptor antagonist; IP-10, human interferon-inducible protein 10; MCP-1, monocyte chemoattractant protein-1; TNF-RI, tumor necrosis factor receptor-1.

BTLA was negatively correlated with IL-1RA (p= 0.014), and TIM-3 was positively correlated with IL-8 (p= 0.017) in all PWH. Interestingly, while BTLA and PD-1 were negatively correlated with IL-1RA (p= 0.018 and p= 0.049, respectively), PD-1 was positively correlated with IP-10 (p= 0.028), and TIM-3 was positively correlated with IL-8 (p= 0.018) in PWH who had persistently liver stiffness (LSM≥ 12.5 kPa). No significant correlations were found in PWH who had no persistently liver stiffness (LSM< 12.5 kPa) (**Figure 3**).

## 4 Discussion

This research is the first to report an association between plasma levels of soluble BTLA, PD-1, and TIM-3 one year after successful HCV therapy and persistently high liver stiffness five years later in PWH.

Despite substantial advances in HCV therapy, information on its impact on the course of liver disease is still scarce, particularly among PWH. After achieving SVR, many studies have shown fibrosis/cirrhosis regression among HCV-infected patients. However, these studies have mostly been performed in the early stage after HCV therapy (25), as the nature of this process is highly dynamic (26), which might vary in the long term. Fibrosis regression has been suggested to reach its plateau about one year after achieving SVR (27), with chronic inflammation due to immune responses considered the most critical driver of liver fibrogenesis and cirrhosis (28). In our study, patients decreased or increased their LSM value during the follow-up, but a non-significant change was observed globally.

We found that soluble BTLA, PD-1, and TIM-3 one year after completion of successful HCV treatment were significantly associated with the presence of persistently liver stiffness five years after treatment (end of follow-up). In addition, we found that PD-1 and TIM-3 at baseline (before HCV treatment) were also significantly associated with persistently elevated liver stiffness. These findings are consistent with previous studies concerning surface proteins. Surface BTLA, PD-1, and TIM-3 are increased in HCV-infected patients who progressed to cirrhosis and HCC (7–9). Chen et al. found higher levels of co-inhibitory molecules, such as surface BTLA and PD-1, in HBV-infected patients who progressed to cirrhosis or HCC (10). Surface PD-1 and TIM-3 are also upregulated in T cells from HCC tissues of HCV-uninfected patients (11).

Although the biological significance of these proteins is not fully understood, they are thought to be products of alternatively spliced variants or shedding from immune cell surface molecules (29). These soluble immune checkpoint proteins may have immunomodulatory effects in the HCV- and HIV-induced immune dysfunction (8, 30), in which permanent activation of T and B cells leads to a state of immune exhaustion. This exhaustion is partially due to activation caused by the following interactions: 1) PD-1/PD-L1: soluble PD-1 can compete with membrane PD-1, reducing inhibitory signaling to T cells, partially restoring immune function and exacerbating inflammatory responses (31); 2) BTLA/HVEM: soluble BTLA can block this interaction, restoring T and B cell proliferation and function and increasing inflammation (32); 3) TIM-3/Galectin-9: soluble TIM-3 can disrupt this interaction, partially alleviating T-cell exhaustion, restoring their ability to proliferate and produce pro-inflammatory cytokines (30). Thus, elevated levels of these soluble molecules may reflect an attempt by the immune system to reverse this exhaustion state, although their impact may be dual: a) protective, by enhancing immune cell function, and b) detrimental, by amplifying inflammation and thus liver injury to cirrhosis (33).

Our study supports that the dysregulation of these proteins and their association with persistently liver stiffness and other pathologies derived from cirrhosis remain in PWH even after HCV eradication. Hence, BTLA, PD-1, and TIM-3 could be predictive biomarkers of persistently liver stiffness and serious pathologies derived from cirrhosis, but further studies are needed to corroborate our findings. Besides, we analyzed the correlation of these immune checkpoints with several inflammatory biomarkers, finding a significant negative correlation between BTLA and PD-1 and the anti-inflammatory IL-1RA; and positive correlations between PD-1 and TIM-3 with pro-inflammatory chemokines (IP-10 and IL-8, respectively) in PWH who had persistently liver stiffness, supporting the immune activation and inflammaging in these patients.

The following limitations should be considered for a correct interpretation of the study: i) the limited sample size could have restricted the detection of other associations of smaller magnitude; ii) different HCV therapies (IFN-based therapy and IFN-free therapy) could have biased the results, although we controlled for this factor by including it as a covariate in the GLM analysis; iii) although all PWH included in the study were on stable ART and had undetectable HIV viral load, the absence of exhaustive control data during follow-up prevents us from completely ruling out the possibility of viral rebounds at any point.

In conclusion, elevated plasma levels of immune checkpoint proteins BTLA, PD-1, and TIM-3 one year after successful HCV treatment were associated with persistently high liver stiffness five years later, suggesting a potential immunopathological role in cirrhosis after HCV eradication in PWH.

## Data Availability

The original contributions presented in the study are included in the article/**Supplementary Material**. Further inquiries can be directed to the corresponding authors.
