# Supplementary material for: Immune checkpoint proteins are associated with persistently high liver stiffness after successful HCV treatment in people with HIV: a retrospective study
Source: Front Immunol. 2024 Dec 17;15:1505864. doi: 10.3389/fimmu.2024.1505864 (PMC11686224; doi:10.3389/fimmu.2024.1505864)
Supplement: Supplementary file 1 [file Table1.docx]

Supplementary Material

# Supplementary Figures and Tables

## Supplementary Table 1

## Baseline characteristics of HIV/HCV-coinfected patients stratified by persistently elevated liver stiffness five years after completion of successful HCV treatment.

|  | **Persistently elevated liver stiffness** | **Not persistently elevated liver stiffness** | ***p*-value** |
| --- | --- | --- | --- |
| **No.** | 24 (61.5%) | 15 (38.5%) |  |
| Age (years) | 51 (48–53) | 51 (48–53) | 0.562 |
| Gender |  |  | **0.036** |
| Male | 16 (66.7%) | 15 (100.0%) |  |
| Female | 8 (33.3%) | 0 (0.0%) |  |
| BMI ((kg/m^2^) | 24.7 (22.3–26.3) | 24.6 (23.3–26.3) | 0.795 |
| Smoker |  |  | 0.659 |
| Never | 3 (12.5%) | 2 (13.3%) |  |
| Previous (>6 months) | 5 (20.8%) | 5 (33.3%) |  |
| Current | 16 (66.7%) | 8 (53.3%) |  |
| Alcohol intake (>50g/day) |  |  | 0.507 |
| Never | 11 (45.8%) | 8 (53.3%) |  |
| Previous (>6 months) | 11 (45.8%) | 7 (46.7%) |  |
| Current | 2 (8.3%) | 0 (0.0%) |  |
| Intravenous drug user |  |  | 0.976 |
| Never | 5 (20.8%) | 4 (26.7%) |  |
| Previous (>6 months) | 19 (79.2%) | 11 (73.3%) |  |
| Current |  |  |  |
| Previous HCV therapy |  |  | 0.999 |
| Yes | 13 (54.2%) | 8 (53.3%) |  |
| No | 11 (45.8%) | 7 (46.7%) |  |
| MASLD (n = 37) |  |  | 0.999 |
| Yes | 5 (22.7%) | 3 (20.0%) |  |
| No | 18 (77.3%) | 12 (80.0%) |  |
| **Liver markers** |  |  |  |
| LSM (kPa) baseline | 27.7 (21.8–36.3) | 13.5 (11.4–26.6) | **0.004** |
| LSM (kPa) 1 year after HCV treatment | 22.8 (20.6–33.9) | 9.0 (7.6–13.9) | **<0.001** |
| LSM (kPa) 5 years after HCV treatment | 23.5 (17.0–33.9) | 7.6 (6.7–9.4) | **<0.001** |
| HSI (n = 37) | 31.7 (28.2–35.4) | 34.3 (33.6–36.4) | 0.127 |
| FIB-4 (n = 37) | 6.7 (2.7–9.7) | 2.9 (2.2–4.3) | 0.056 |
| **HCV markers** |  |  |  |
| HCV genotype (n = 37) |  |  | 0.534 |
| 1 | 16 (69.6%) | 12 (85.7%) |  |
| 3 | 4 (17.4%) | 1 (7.1%) |  |
| 4 | 3 (13.0%) | 1 (7.1%) |  |
| Log_10_ HCV-RNA (IU/mL) | 6.0 (5.4–6.4) | 6.3 (5.8–6.7) | 0.113 |
| HCV-RNA > 850.000 IU/mL |  |  | 0.340 |
| Yes | 13 (54.2%) | 9 (60.0%) |  |
| No | 11 (45.8%) | 6 (40.0%) |  |
| **HCV therapy** |  |  | **0.007** |
| pegIFN | 9 (37.5%) | 13 (86.7%) |  |
| DAAs | 15 (62.5%) | 2 (13.3%) |  |
| **HIV markers** |  |  |  |
| Previous AIDS (n = 38) |  |  | 0.782 |
| Yes | 0 (0.0%) | 1 (7.1%) |  |
| No | 24 (100.0%) | 13 (92.9%) |  |
| CD4+ T-cells/mm^3^ | 372.0 (245.3–518.5) | 515.0 (367.0–772.5) | 0.172 |
| CD4+ T-cells < 500 cells/mm^3^ |  |  | 0.147 |
| Yes | 18 (75.0%) | 7 (46.7%) |  |
| No | 6 (25.0%) | 8 (53.3%) |  |
| **HIV antiretroviral therapy** |  |  |  |
| NRTI + NNRTI | 9 (37.5%) | 2 (13.3%) | 0.102 |
| NRTI + II | 9 (37.5%) | 6 (40.0%) |  |
| NRTI + PI | 1 (4.2%) | 5 (33.3%) |  |
| PI+II+NNRTI/MVC | 1 (4.2%) | 0 (0.0%) |  |
| Others | 4 (16.7%) | 2 (13.3%) |  |

Statistics: The values are expressed as the absolute number (percentage) and median (interquartile range). P-values were calculated by the Chi-square test and the Mann-Whitney U test. Abbreviations: HCV, hepatitis C virus; HIV, human immunodeficiency virus; BMI, body mass index; MASLD, metabolic dysfunction-associated steatotic liver disease; LSM, liver stiffness measurement (before treatment); kPa, kilopascal; HSI, hepatic steatosis index; FIB-4, fibrosis-4; pegIFN, pegylated interferon; DAAs, direct-acting antivirals; AIDS, acquired immune deficiency syndrome; NRTI, nucleoside analogue HIV reverse transcriptase inhibitor; NNRTI, non-nucleoside analogue HIV reverse transcriptase inhibitor; II, HIV integrase inhibitor; PI, HIV protease inhibitor.

## Supplementary Table 2

## Association of plasma immune checkpoint proteins one year after completion of successful HCV treatment with persistently elevated liver stiffness (LSM ≥12.5 kPa) at the end of follow-up (five years after treatment) in HIV/HCV-coinfected patients.

|  | **Un-adjusted** | | | **Adjusted** | |  |
| --- | --- | --- | --- | --- | --- | --- |
| **Marker** | **AMR (95%CI)** | ***p*-value** | ***q*-value** | **aAMR (95%CI)** | ***p*-value** | ***q*-value** |
| BTLA | 1.42 (1.04–1.95) | **0.034** | **0.094** | 1.49 (1.15–1.96) | **0.006** | **0.051** |
| CD137(4-1BB) | 1.23 (0.87–1.73) | 0.250 | 0.472 | 1.05 (0.71–1.55) | 0.827 | 0.891 |
| CD152(CTLA4) | 1.19 (0.88–1.63) | 0.270 | 0.472 | 1.39 (1.00–1.90) | 0.051 | 0.143 |
| CD27 | 1.04 (0.82–1.33) | 0.719 | 0.774 | 1.13 (0.88–1.46) | 0.337 | 0.473 |
| CD28 | 1.12 (0.86–1.48) | 0.391 | 0.548 | 1.16 (0.91–1.48) | 0.233 | 0.362 |
| CD80 | 1.71 (1.25–2.35) | **0.002** | **0.028** | 1.40 (0.99–1.97) | 0.067 | 0.157 |
| GITR | 1.16 (0.84–1.59) | 0.376 | 0.548 | 1.20 (0.91–1.60) | 0.207 | 0.361 |
| HVEM | 0.95 (0.73–1.23) | 0.703 | 0.774 | 0.99 (0.78–1.27) | 0.965 | 0.965 |
| IDO | 0.95 (0.57–1.57) | 0.833 | 0.833 | 0.46 (0.22–1.00) | 0.051 | 0.143 |
| LAG-3 | 1.27 (1.05–1.55) | **0.022** | **0.088** | 1.06 (0.84–1.33) | 0.653 | 0.762 |
| PD-1 | 1.45 (1.06–1.97) | **0.025** | **0.088** | 1.49 (1.14–1.97) | **0.007** | **0.051** |
| PD-L1 | 1.06 (0.87–1.29) | 0.559 | 0.712 | 1.14 (0.95–1.37) | 0.157 | 0.315 |
| PD-L2 | 1.29 (1.05–1.59) | **0.021** | **0.088** | 1.09 (0.87–1.37) | 0.461 | 0.587 |
| TIM-3 | 1.24 (1.01–1.51) | **0.047** | **0.109** | 1.28 (1.05–1.56) | **0.020** | **0.092** |

Statistics: Data were calculated by Generalized Linear Models (GLM) with a gamma distribution (log-link). Multivariable models were adjusted by age, gender, HCV treatment (IFN-based therapy or DAAs), LSM at one year after treatment, and time elapsed between the two times, which were previously selected by a stepwise method (forward) (see Results Section). The q-values represent p-values corrected for multiple testing using the False Discovery Rate (FDR; Benjamini and Hochberg procedure). Statistically significant differences are shown in bold. Abbreviations: AMR, arithmetic mean ratio; aAMR, adjusted AMR; 95%CI, 95% of confidence interval; p, level of significance; q, corrected level of significance; BTLA, B and T lymphocyte attenuator; CD, cluster of differentiation; GITR, glucocorticoid-induced TNFR-related; HVEM, herpesvirus entry mediator; IDO, indoleamine 2,3-dioxygenase; LAG-3, lymphocyte activation gene-3; PD-1, programmed cell death protein 1; PD-L1, programmed death-ligand 1; PD-L2, programmed death-ligand 2; TIM-3, T-cell immunoglobulin and mucin-domain containing-3.

## Supplementary Table 3

## Association of plasma immune checkpoint proteins at baseline (before HCV treatment) with persistently elevated liver stiffness (LSM ≥12.5 kPa) at the end of follow-up (five years after treatment) in HIV/HCV-coinfected patients.

|  | **Un-adjusted** | | | **Adjusted** | |  |
| --- | --- | --- | --- | --- | --- | --- |
| **Marker** | **AMR (95%CI)** | ***p*-value** | ***q*-value** | **aAMR (95%CI)** | ***p*-value** | ***q*-value** |
| BTLA | 2.19 (1.11–4.31) | **0.030** | **0.045** | 1.60 (0.85–3.01) | 0.154 | 0.154 |
| PD-1 | 2.71 (1.27–5.78) | **0.014** | **0.042** | 2.71 (1.27–5.78) | **0.014** | **0.042** |
| TIM-3 | 1.26 (1.01–1.58) | **0.048** | **0.048** | 1.26 (1.01–1.58) | **0.048** | **0.072** |

Statistics: Data were calculated by Generalized Linear Models (GLM) with a gamma distribution (log-link). Multivariable models were adjusted by age, gender, LSM at baseline, and time elapsed between the two times, which were previously selected by a stepwise method (forward) (see Results Section). The q-values represent p-values corrected for multiple testing using the False Discovery Rate (FDR; Benjamini and Hochberg procedure). Statistically significant differences are shown in bold. Abbreviations: AMR, arithmetic mean ratio; aAMR, adjusted AMR; 95%CI, 95% of confidence interval; p, level of significance; q, corrected level of significance; BTLA, B and T lymphocyte attenuator; PD-1, programmed cell death protein 1; TIM-3, T-cell immunoglobulin and mucin-domain containing-3.
